# Leisure-time physical activity, sedentary behaviors, sleep, and cardiometabolic risk factors at baseline in the PREDIMED-PLUS intervention trial: A cross-sectional analysis

**DOI:** 10.1371/journal.pone.0172253

**Published:** 2017-03-08

**Authors:** Nuria Rosique-Esteban, Andrés Díaz-López, Miguel A. Martínez-González, Dolores Corella, Albert Goday, J. Alfredo Martínez, Dora Romaguera, Jesus Vioque, Fernando Arós, Antonio Garcia-Rios, Francisco Tinahones, Ramon Estruch, José Carlos Fernández-García, José Lapetra, Luís Serra-Majem, Xavier Pinto, Josep A. Tur, Aurora Bueno-Cavanillas, Josep Vidal, Miguel Delgado-Rodríguez, Lidia Daimiel, Clotilde Vázquez, Miguel Ángel Rubio, Emilio Ros, Jordi Salas-Salvadó, PREDIMED-PLUS investigators

**Affiliations:** 1 Human Nutrition Unit, University Hospital of Sant Joan de Reus, Department of Biochemistry and Biotechnology, Pere Virgili Institute for Health Research, Rovira i Virgili University, Reus, Spain; 2 CIBER de Fisiopatología de la Obesidad y la Nutrición (CIBEROBN), Instituto de Salud Carlos III, Madrid, Spain; 3 Department of Preventive Medicine and Public Health, University of Navarra-Navarra Institute for Health Research, Pamplona, Spain; 4 Department of Preventive Medicine, University of Valencia, Valencia, Spain; 5 Servicio de Endocrinología, Hospital del Mar, Barcelona, Departament de Medicina, Universitat Autònoma de Barcelona, Barcelona, Spain; 6 Department of Nutrition, Food Sciences, and Physiology, Center for Nutrition Research, University of Navarra, Pamplona, Spain; 7 Instituto de Investigación Sanitaria de Palma (IdISPa), University Hospital of Son Espases, Palma de Mallorca, Spain; 8 University of Miguel Hernández, Alicante, Spain; CIBER de Epidemiología y Salud Pública (CIBERESP), Instituto de Salud Carlos III, Madrid, Spain; 9 Department of Cardiology, University Hospital Araba, Vitoria, Spain; 10 Lipids and Atherosclerosis Unit, Department of Internal Medicine, Reina Sofia University Hospital, IMIBIC, University of Córdoba, Córdoba, Spain; 11 Department of Endocrinology and Nutrition, Virgen de la Victoria Hospital, Malaga University, Malaga, Spain; 12 Department of Internal Medicine, Hospital Clínic, IDIBAPS August Pi i Sunyer Biomedical Research Institute, University of Barcelona, Barcelona, Spain; 13 Facultad de Ciencias de la Educación, Instituto de Investigación Biomédica de Málaga (IBIMA), Universidad de Málaga, Málaga, Spain; 14 Department of Family Medicine, Distrito Sanitario Atencion Primaria Sevilla, Sevilla, Spain; 15 Research Institute of Biomedical and Health Sciences, University of Las Palmas de Gran Canaria, Las Palmas de Gran Canaria, Spain; 16 Lipid Unit, Department of Internal Medicine, Bellvitge Biomedical Research Institute (IDIBELL)-Hospital Universitari de Bellvitge, L'Hospitalet de Llobregat, Barcelona, Spain; 17 Research Group on Community Nutrition and Oxidative Stress, University of the Balearic Islands, Palma de Mallorca, Spain; 18 Departament of Preventive Medicine and Public Health, University of Granada; CIBER de Epidemiología y Salud Pública (CIBERESP), Instituto de Salud Carlos III, Madrid, Spain; 19 Department of Endocrinology and Nutrition, Hospital Clínic, Barcelona, Spain; CIBER de Diabetes y Enfermedades Metabólicas Asociadas (CIBERDEM), Instituto de Salud Carlos III, Madrid, Spain; 20 Department of Health Sciences, University of Jaen; Jaen, Spain; CIBERESP, Instituto de Salud Carlos III, Madrid, Spain; 21 Department of Cardiovascular Epidemiology and Population Genetics, Centro Nacional de Investigaciones Cardiovasculares (CNIC), Madrid, Spain; Madrid Institute for Advanced Studies (IMDEA) Food Institute, Madrid, Spain; 22 Department of Endocrinology and Nutrition, University Hospital Fundación Jiménez Díaz, Madrid, Spain; 23 Endocrinology and Nutrition Department, Hospital Clínico San Carlos-IdISSC, Madrid, Spain; 24 Department of Lipids, Hospital Clínic, Institut d'Investigacions Biomediques August Pi Sunyer (IDIBAPS), University of Barcelona, Barcelona, Spain; Indiana University Richard M Fairbanks School of Public Health, UNITED STATES

## Abstract

Limited data exists on the interrelationships between physical activity (PA), sedentary behaviors and sleep concerning cardiometabolic risk factors in aged adults at high cardiovascular disease risk. Our aim was to examine independent and joint associations between time spent in leisure-time PA, sedentary behaviors and sleep on the prevalence of obesity, type 2 diabetes (T2D) and components of the metabolic syndrome (MetS) in Mediterranean individuals at high cardiovascular risk. Cross-sectional analyses were performed on baseline data from 5776 Spanish adults (aged 55-75y in men; 60-75y in women) with overweight/obesity and MetS, from October 2013 to October 2016, in the PREDIMED-PLUS trial. Employing multivariable-adjusted Cox regression with robust variance and constant time (given the cross-sectional design), higher prevalence of obesity, T2D and abdominal obesity as component of the MetS were associated with greater time in TV-viewing (Relative Risk, RR: 1.02, 95%CI: 1.01, 1.03; RR:1.04, 95%CI: 1.02, 1.06 and RR: 1.01 95%CI: 1.00, 1.02; respectively, all *P* < .01). Conversely, greater time in moderate-vigorous PA (MVPA) was associated with lower prevalence of obesity, T2D, abdominal obesity and low HDL-cholesterol (RR: 0.95, 95%CI: 0.93, 0.97; RR: 0.94, 95%CI: 0.89, 0.99; RR: 0.97, 95%CI: 0.96, 0.98; and RR: 0.95, 95%CI: 0.91, 0.99, respectively, all *P* < .05). For these outcomes, theoretically substituting 1-h/day of MVPA for 1-h/day TV-viewing was also significantly associated with lower prevalence (RR 0.91 to 0.97, all *P* < .05). Similar lower RR in these outcomes was observed when substituting 1-h/day of MVPA for 1-h/day of sleeping. Longer time watching TV and not meeting MVPA recommendations were jointly associated with higher RR of the prevalence of obesity and T2D. We concluded that, in senior individuals at high cardiovascular risk, greater time spent on MVPA and fewer on sedentary behaviors was inversely associated with prevalence of obesity, T2D, and some of the components of MetS.

## Introduction

Physical activity (PA), sedentary behaviors and sleeping time are main components of the circadian cycle of which research has shown to strongly impact human health and to relate with multiple cardiometabolic risk factors [1–3]. It is well established that increasing time spent in MVPA is associated to a substantial risk reduction in cardiovascular disease (CVD) and mortality [4–6]. Contrary, sedentary behaviors including sitting or reclining postures and activities with ≤1.5 metabolic equivalent task (MET) of energy expenditure [7], such as watching TV, have been repeatedly linked to increased CVD morbimortality [8–10], as well as the development of T2D and MetS [11–13]. The fact these observations have been consistently reported when controlling for physical activity suggests that sedentary behaviors may act as different construct than physical inactivity [4,14] The relationships between sleep duration and cardiometabolic conditions are complex, yet sleep deprivation (i.e. sleeping ≤7 h, a common practice among the general population) has been consistently related to higher average weight gain [15], and to higher risk of obesity [16,17], T2D [18,19], hypertension and other hormonal and metabolic disturbances [19].

Importantly, these cardiometabolic health-related effects attributed to PA, sedentary behaviors and altered sleep patterns have been extensively reported when assessed independently to each other [11,12,20,21]. However, time within the circadian cycle is finite, which challenges the assumption that a sole activity acts independently from the others, and suggests that the time spent in one activity is intrinsically co-dependent of the time spent on the rest of the activities comprising total day time [22]. Isotemporal substitution modeling enables to better discriminate the effects on health outcomes of simultaneously performing a single activity (e.g. MVPA) and displacing another (e.g. watching TV), while controlling for other day activities and capturing the effect of time [23,24]. These advantages may be especially important from a public health perspective given that combined strategies, such as engaging in more MVPA and less sedentary behaviors may be effective to prevent obesity, T2D and other relevant cardiometabolic risk factors [4].

To date, some cross-sectional [22,25–27] and prospective studies [28–30] have addressed the interrelationships between PA, sedentary behaviors and sleep in relation to cardiometabolic risk factors in different populations including overweight/obese persons [22,28], T2D patients [30] and healthy individuals [25,26]. Nevertheless, these associations have been barely explored in a large population of elderly adults at high CVD risk—a typically overweight, sedentary and physically inactive group. Considering the high prevalence of cardiometabolic disorders in this sector of the population and their consequent higher risks for several chronic diseases, such studies are warranted in order to contribute to the development of preventive strategies. Therefore, the aim of the present study was to examine the independent and combined associations between time spent in different activities, such as leisure-time PA, TV-viewing (as a proxy for sedentary behaviors) and sleep in relation to relevant cardiometabolic risk factors including obesity, T2D and individual components of the MetS in senior adults at high CV risk. By using isotemporal substitution modeling [31], the associations of theoretically replacing one type of activity for another with the same amount of time on the prevalence of the study outcomes were also evaluated.

## Materials and methods

### Study design and participants

The present investigation is a cross-sectional analysis on baseline data within the frame of the PREDIMED-PLUS study, a 6-year multicenter, randomized, parallel-group, primary prevention clinical trial conducted in Spain to assess the effect on CVD morbimortality of an intensive weight loss intervention program based on an energy-restricted traditional Mediterranean diet, PA promotion and behavioral support, in comparison with an usual care intervention only with energy-unrestricted Mediterranean diet (control group). A more detailed description of the PREDIMED-PLUS study is available at http://predimedplus.com/. This study was registered at the International Standard Randomized Controlled Trial (ISRCT; http://www.isrctn.com/ISRCTN89898870) with number 89898870. Registration date: 24 July 2014.

From October 2013 to October 2016, a total of 5776 participants were recruited and randomized in 22 centres from different universities, hospitals and research institutes of Spain. Each of these centres recruited participants from several Primary Care Health Facilities belonging to the National Health System. The eligible participants were community-dwelling adults (aged 55–75 in men; 60–75 in women) with overweight/obesity [body mass index (BMI) ≥27 and <40 kg/m^2^], who met at least three components of the MetS according to the updated harmonized criteria of the International Diabetes Federation and the American Heart Association and National Heart, Lung and Blood Institute [32]. All participants included in the current analysis presented data on PA, sedentary behaviors and sleeping time.

All participants provided written informed consent, and the study protocol and procedures were approved according to the ethical standards of the Declaration of Helsinki by all the participating institutions: CEI Provincial de Málaga, CEI de los Hospitales Universitarios Virgen Macarena y Virgen del Rocío, CEI de la Universidad de Navarra, CEI de las Illes Balears, CEIC del Hospital Clínic de Barcelona, CEIC del Parc de Salut Mar, CEIC del Hospital Universitari Sant Joan de Reus, CEI del Hospital Universitario San Cecilio, CEIC de la Fundación Jiménez Díaz, CEIC Euskadi, CEI en Humanos de la Universidad de Valencia, CEIC del Hospital Universitario de Gran Canaria Doctor Negrín, CEIC del Hospital Universitario de Bellvitge, CEI de Córdoba, CEI de Instituto Madrileño De Estudios Avanzados, CEIC del Hospital Clínico San Carlos, CEI Provincial de Málaga, CEI de las Illes Balears, CCEI de la Investigación Biomédica de Andalucía and CEIC de León.

### Exposure variables

Sedentary behaviours were evaluated on weekdays and weekends with the validated Nurses’ Health Study questionnaire for sedentary behaviours [33], consisting of a set of open-ended questions assessing the average daily time spent over the last year in watching TV, sitting while using computer, sitting on journeys (for work purposes or leisure time, as driver or passenger car, subway, bus, etc) and total sitting. Answers included 12 categories ranging from 0 to ≥9 h/day of sitting time for the corresponding activity. Because TV time is the most prevalent sedentary behavior, for which previous investigations among aged population have suggested to fairly capture total sedentary time [34] and to consistently associate to higher risk of various cardiometabolic risk factors and cardiovascular mortality in a dose-response fashion [8], the present study has evaluated TV time as a proxy for sedentary behaviors.

Leisure-time PA was assessed using the validated REGICOR questionnaire [35] (including questions to collect information the type of activity, frequency (number of days) and duration (min/day). The intensity was assigned based on the compendium of PA [36]. A trained interviewer collected the required information about 6 types of activities performed during a conventional month: brisk walking (5 MET), walking at a slow/normal pace (4 MET), walking in the countryside (6 MET), climbing stairs (7 MET), working in the garden (5 MET), exercise or play sports at home, outdoors or in a gym (11 MET). According to PA intensity, activities were categorized into light PA <4.0 MET, moderate PA 4–5.5 MET and vigorous PA ≥6.0 MET. Total leisure-time PA-related energy expenditure was estimated as the summed product of frequency, duration and intensity of each activity divided by 30 days/month (MET·min/day). For the present study, leisure-time PA was categorized in light PA (including leisurely stroll or walk) and MVPA (including the sum for any activity of moderate or greater intensity). Finally, PA time was computed as the sum of frequency*duration of each activity divided by 30 to obtain the number of min/day.

Regarding sleep, participants reported their average daily sleeping time for both weekdays and weekends, using the non-validated open question “How many hours do you sleep on average per day on weekdays and weekends?”

### Outcomes ascertainment

Study outcomes were obesity, T2D and individual components of the MetS. Obesity was defined as BMI ≥30 kg/m^2^. T2D was defined as previous clinical diagnosis of diabetes, or HbA1c levels ≥6.5% or use of antidiabetic medication at baseline. Individual components of the MetS were defined as follows: abdominal obesity (waist circumference ≥102 cm in men; ≥88 cm in women), high blood pressure (systolic and/or diastolic ≥130/85 mmHg or using antihypertensive drugs), hyperglycaemia (glucose ≥5.5 mmol/L or taking medication for elevated glucose), hypertriglyceridemia (triglycerides ≥1.7 mmol/L or taking triglyceride-lowering medication), low HDL-cholesterol (HDL-c <1.03 mmol/L in men and <1.3 mmol/L in women or taking HDL-c raising medication) [32].

### Covariate assessment

The covariates were evaluated using self-reported questionnaires about socio-demographic factors (sex, age, education, and marital and employment status), smoking habits, personal and family history of illness, medical conditions, medication use and a 17-item screening questionnaire assessing adherence to an energy-restricted Mediterranean diet.

Anthropometric variables and blood pressure were determined by trained staff and in accordance with the PREDIMED-PLUS operations protocol. Weight and height were measured with calibrated scales and a wall-mounted stadiometer, respectively. BMI was calculated as the weight in kilograms divided by the height in meters squared. Waist circumference was measured midway between the lowest rib and the iliac crest, after normal exhalation, using an anthropometric tape. Blood pressure was measured in triplicate with the use of a validated semiautomatic oscillometer (Omron HEM-705CP, Netherlands) while the participant was in a seated position after 5 minutes of rest.

Blood samples were collected after 12 hours overnight fast and biochemical analyses were performed on fasting plasma glucose, HDL-c and triglycerides concentrations in local laboratories using standard enzymatic methods.

### Statistical analyses

In order to provide with more detailed information, baseline characteristics are presented according to categories of total leisure-time PA in min/day (<15, from 15 to < 30, from 30 to < 60, from 60 to < 120 and ≥120) as means ± SD and number (%) by using one-way ANOVA or chi-square tests as appropriate.

Given the cross-sectional design, Cox regression models with constant time of follow-up for all individuals and robust variance estimates were fitted to estimate RR and 95% confidence intervals (CI) for each study outcome (obesity, T2D, and individual components of the MetS, all as dichotomous variables) per 1-h/day increase in time spent in each activity separately (TV-viewing, light PA and MVPA and sleeping, all as continuous variables). Correspondingly, the time *t* was set to a constant (*t* = 1). According to methodologists, this model is better suited than logistic regression for cross-sectional studies with frequent prevalent outcomes, such as the present study, since it avoids the overestimation of the prevalence ratios derived from the odds ratios when logistic regression is applied in analysis with frequent outcomes [37,38].

A crude model and three multivarible-adjusted Cox regression models were fitted as follows: a) model 1 [adjusted for age (continuous), sex, education level (illiterate/primary education, secondary education and academic/graduate), smoking status (never smoker, past smoker and current smoker), marital status (single/divorced, married and widower), family history of coronary heart disease (yes or no) and energy-restricted Mediterranean diet adherence (score 0 to 17 items, in categories of <8 or ≥9 items)], b) model 2 [model 1 plus the time spent on the rest of the activities to precisely assess the independent effect of an activity]and c) model 3 [model 2 plus each of the other components of the MetS, only for the associations with each component of the MetS]. All models were stratified by recruiting center. In order to correct for multiple testing, the Benjamini-Hochberg approach was applied to calculate false discovery rate q values [39]. Effect modification by sex, age (≤65, >65 years) and the exposure variables (time spent in sleeping time, TV-viewing, light PA and MVPA) on each outcome was evaluated by calculating te likelihood ratio test between the fully adjusted model and the same model adding the interaction product-term. All analysis testing for effect modifications by sex and age showed no statistical significance (*P* >.40 for all interactions).

Taking advantage of the interpretation and the relevance to public health recommendations, we employed isotemporal substitution modeling to estimate the theoretical association of replacing 1-h/day from one activity for 1-h/day of another activity on the prevalence of each outcome, adjusting for potential confounders as detailed previously in models 2 and 3. For these analyses, all activity variables (e.g., time spent in TV-viewing, light PA, and MVPA), except the activity of interest which was dropped (e.g., sleeping time), were entered simultaneously into the models, along with a total discretionary time and covariates as follows: *h*(*t*) = *h*_*0*_(t) exp [β_1_(TV-viewing) + β_2_(light PA) + β_3_(MVPA) + β_4_(total discretionary time) + β_5_(covariates)], where *t* = 1. Total discretionary time was computed as a result of the sum of hours spent in TV-viewing, light PA, MVPA and sleeping. Therefore, it is assumed that the model was isotemporal when including the total discretionary time variable herein. Thus, the Cox regression estimates for the included activities variables reflects the RR for each outcome observed when the time spent in these activities increases 1-h/day because the time spent in the omitted activity (e.g., sleeping) decreases 1-h/day.

Finally, because PA and TV-viewing are two closely-related lifestyle behaviours concerning to cadiometabolic outcomes such as obesity and T2D [40] we explored the joint associations of combining MVPA and time spent watching TV on obesity and T2D. For this purpose, MVPA was first dichotomized into meeting or not meeting current WHO recommendations [41] for MVPA set in ≥2.5 h/week (yes/no). Time spent in watching TV (in hours) was categorized in three approximately equally distributed groups: low TV (≤2h/day), medium TV (>2 to ≤4h/day) and high TV (>4h/day). Therefore, each participant was cross-allocated to one of the six joint categories and meeting MVPA recommendations and low TV group was considered as the reference category. The interaction between meeting or not meeting the recommendations for MVPA and time spent watching TV in their associations with each outcome was examined by calculating the likelihood ratio test between the fully adjusted model and the same model including the interaction product-term (*P*>.30 for all the interactions).

Significance for all statistical tests was *P* < .05 for bilateral contrast. All analyses were cross-sectional, and performed using Stata (14.0, StataCorp LP, Tx. USA).

## Results

Individuals undergoing screening and meeting inclusion criteria, but eventually not being randomized (n = 2239); and the trial participants randomized (n = 5776) showed no statistically significant differences in terms of sex, age and BMI. Trial participants mean age was 65±4.9y and 51.9% were men. More than 60% of the participants reported sleeping between 7 to <9 h/day. On average, individuals spent 4.9 ± 2.3 h/day sitting and their average time watching TV was 3.3±1.7 h/day, suggesting that they dedicate most of their sitting time watching TV (67%). The total mean time spent in leisure time PA was 66.8 min/day and 45% of the population reported spending ≥60 min/day on PA. MVPA was the most frequent PA intensity in this population, contributing to 60% of the total min/ day. Table 1 presents participant characteristics according to categories of total daily leisure-time PA. Compared to less active individuals, physically active participants were more likely to be men, had lower BMI, lower body weight and waist circumference, and spent less daily time in sedentary behaviors and watching TV. They also had greater daily energy expenditure from PA and were more likely to adhere to the energy-restricted Mediterranean diet.

**Table 1:** Baseline characteristics of the participants in the PREDIMED-PLUS intervention trial (n = 5776).

	Total leisure time physical activity (min/day)					*P* ^¶^
	< 15	15 to <30	30 to <60	60 to <120	≥ 120	
	n = 861	n = 817	n = 1558	n = 1695	n = 845	
**Age, years**	64.6±4.9	64.6±5.2	65.0±5.0	65.3±4.8	65.1±4.7	.002
**Men, n (%)**	361(41)	368(45)	738(47)	924(54)	610(72)	< .001
**BMI, kg/m**^**2**^	33.6±3.6	33.0±3.49	32.6±3.5	32.1±3.3	31.9±3.1	< .001
**Weight, kg**	88.6±13.1	87.1±13.4	86.2±13.1	85.5±12.7	87.2±11.9	< .001
**Waist circumference, cm**	109.7±9.8	108.3±10.1	107.2±9.5	106.6±9.3	107.6±8.7	< .001
**Married status, n (%)**						< .001
Single or divorced	119(14)	121(15)	207(13)	196(12)	96(11)	
Married	642(74)	597(73)	1169(75)	1323(78)	692(81)	
Widower	100(11)	99(12)	184(11)	176(10)	57(7)	
**Education level, n (%)**						.689
Illiterate or primary education	437(51)	400(49)	779(50)	841(49)	443(52)	
Secondary education	241(28)	250(30)	443(28)	501(30)	247(29)	
Academic or graduate	183(21)	167(20)	336(22)	353(21)	155(18)	
**Smoking habit, n (%)**						< .001
Never smoked	393 (46)	392(48)	721(46)	751(44)	305(36)	
Former smoker	348(40)	307(37)	666(43)	749(44)	450(53)	
Current smoker	120(14)	118(14)	171(11)	195(12)	90(11)	
**Sedentary time, h/day**	5.4±2.6	5.2±2.5	5.0±2.3	4.6±2.1	4.3±1.9	< .001
**TV-viewing time, h/day**	3.5±1.9	3.3±1.7	3.3±1.7	3.1±1.7	2.9±1.6	< .001
**Habitual sleeping time, h/day**	7.0±1.3	7.0±1.3	6.9±1.2	7.1±1.2	7.1±1.2	.232
**Leisure time physical activity, MET.min/day**	29.3±27.9	118.8±45.8	244.8±90.5	464.9±157.2	940.9±387.0	< .001
**Current medication use, n (%)**						
Antihypertensive agents	679(79)	637(78)	1216(78)	1324(78)	668(79)	.965
Hypolipidemic agents ^1^	17(2)	28(3)	37(2)	60(3)	25(3)	.114
**Familiar history of coronary heart disease, n (%)**	152(18)	135(17)	274(18)	255(15)	154(18)	.188
**Prevalence of type 2 diabetes, n (%)**	276(32)	269(32)	499(32)	520(31)	268(31)	.825
**Systolic blood pressure, mmHg**	138.9±17.5	138.6±17.9	139.2±16.9	140.7±16.7	141.6±16.5	.055
**Diastolic blood pressure, mmHg**	80.7±10.1	80.7±10.2	80.8±9.8	80.6±9.7	81.0±10.3	.830
**Plasma fasting glucose, mmol/L**	6.4±1.8	6.4±1.7	6.5±1.8	6.4±1.7	6.5±1.7	.598
**Plasma triglycerides, mmol/L**	1.8±0.9	1.7±0.8	1.8±0.9	1.7±0.8	1.7±0.8	.059
**Plasma HDL-c, mmol/L**	1.2±0.3	1.2±0.3	1.2±0.2	1.2±0.3	1.2±0.3	.117
**Adherence to Mediterranean diet(score 0 to 17 items)**	7.7±2.6	8.2±2.6	8.3±2.7	8.7±2.7	8.9±2.6	< .001

Data is presented as mean ± SD unless otherwise indicated. Abbreviations: BMI, Body mass index; HDL-c, High-density lipoprotein-cholesterol; MET, metabolic equivalent of task.

^1^Hypolipidemic medication included use of fibrate agents. Pearson’s chi-square test for categorical variables and one-factor ANOVA for continuous variables.

^¶^ *P*-value for global comparisons between categories

Table 2 shows that 1-h/day increase in TV-viewing was significantly associated with higher prevalence of obesity (RR: 1.02, 95%CI: 1.01, 1.03) and T2D (RR: 1.04, 95%CI: 1.02, 1.06) after adjustment for the potential confounders and independently of time spent in other activities. Conversely, 1-h/day increase in MVPA was significantly associated with 5 and 6% lower prevalence of obesity and T2D. Even after adjustment for T2D in the model with obesity as the exposure, and *vice versa*, results remained unchanged.

**Table 2:** RR (95%CI) for cardiometabolic risk factors per 60-min/day greater time sleeping, watching TV and PA (n = 5776).

Cardiometabolic risk factors	Sleeping	*P* ^¶^	*q*^^^	TV-viewing	*P* ^¶^	*q*^^^	Light PA^a^	*P* ^¶^	*q*^^^	MVPA^a^	*P* ^¶^	*q*^^^
**Obesity**												
Cases = 4256												
Crude model	0.99(0.97,1.01)	.462	.038	1.03(1.02,1.04)**	< .001	.002	0.99(0.96,1.02)	.365	.030	0.94(0.92,0.96)**	< .001	.004
Multivariable model 1^b^	0.99(0.97,1.01)	.665	.043	1.03(1.02,1.04)**	< .001	.002	0.99(0.96,1.02)	.480	.038	0.95(0.93,0.97)**	< .001	.004
Multivariable model 2^c^	0.99(0.98,1.00)	.659	.041	1.02(1.01,1.03)**	< .001	.002	0.98(0.95,1.01)	.144	.029	0.95(0.93,0.97)**	< .001	.004
**Type 2 diabetes**												
Cases = 1832												
Crude model	1.00(0.97,1.03)	.682	.043	1.05(1.03,1.07)**	< .001	.005	1.06(0.99,1.13)	.075	.021	0.97(0.93,1.02)	.321	.029
Multivariable model 1^b^	1.00(0.97,1.03)	.930	.046	1.05(1.03,1.07)**	< .001	.005	1.04(0.97,1.11)	.214	.030	0.93(0.88,0.98)**	.009	.013
Multivariable model 2^c^	1.00(0.97,1.03)	.918	.046	1.04(1.02,1.06)**	< .001	.005	1.03(0.96,1.10)	.401	.036	0.94(0.90,0.98)*	.032	.014
**Abdominal obesity**												
Cases = 5377												
Crude model	1.00(0.99,1.01)	.842	.050	1.02(1.01,1.03)**	< .001	.007	1.00(0.99,1.01)	.719	.046	0.96(0.95,0.97)**	< .001	.009
Multivariable model 1^b^	1.00(0.99,1.01)	.528	.041	1.01(1.00,1.02)**	< .001	.007	1.00(0.99,1.01)	.386	.036	0.97(0.96,0.98)**	< .001	.009
Multivariable model 2^c^	1.00(0.99,1.01)	.549	.038	1.01(1.00,1.02)**	< .001	.007	1.00(1.98,1.02)	.988	.048	0.97(0.96,0.98)**	< .001	.009
Multivariable model 3^d^	1.00(0.99,1.01)	.567	.036	1.01(1.00,1.02)**	< .001	.007	0.99(0.97,1.01)	.921	.046	0.97(0.96,0.98)**	< .001	.009
**High blood pressure**												
Cases = 4308												
Crude model	1.01(0.99,1.03)	.111	.023	1.00(0.99,1.01)	.601	.039	1.01(0.98,1.04)	.449	.036	1.03(1.01,1.05)**	< .001	.011
Multivariable model 1^b^	1.01(0.99,1.03)	.308	.032	1.00(0.99,1.01)	.669	.045	1.00(0.98,1.02)	.964	.048	1.01(0.99,1.03)	.143	.025
Multivariable model 2^c^	1.00(0.99,1.01)	.301	.030	1.00(0.99,1.01)	.564	.039	1.00(0.97,1.03)	.847	.045	1.01(0.99,1.03)	.122	.021
Multivariable model 3^d^	1.00(0.99,1.01)	.296	.029	1.00(0.99,1.01)	.623	.039	1.00(0.97,1.03)	.835	.043	1.01(0.98,1.04)	.103	.018
**Hyperglycemia**												
Cases = 3980												
Crude model	1.00(0.99,1.01)	.768	.048	1.00(0.99,1.01)	.428	.034	1.03(1.00,1.06)*	.025	.018	0.99(0.97,1.01)	.656	.041
Multivariable model 1^b^	1.00(0.98,1.02)	.992	.050	1.01(0.99,1.03)	.337	.034	1.02(0.99,1.05)	.074	.021	0.98(0.95,1.01)	.057	.018
Multivariable model 2^c^	1.00(0.99,1.01)	.992	.050	1.02(0.99,1.05)	.127	.023	1.02(0.99,1.05)	.127	.025	0.98(0.96,1.01)	.101	.019
Multivariable model 3^d^	1.00(0.98,1.02)	.993	.048	1.00(0.99,1.01)	.515	.034	1.02(0.99,1.05)	.122	.019	0.98(0.96,1.01)	.138	.021
**Hypertriglyceridemia**												
Cases = 2525												
Crude model	1.02(0.99,1.05)	.167	.025	1.02(1.00,1.04)	.056	.019	1.01(0.96,1.06)	.699	.045	0.94(0.90,0.98)**	.001	.014
Multivariable model 1^b^	1.02(1.00,1.04)	.110	.023	1.02(1.00,1.04)*	.020	.016	1.02(0.97,1.07)	.482	.039	0.95(0.91,0.99)**	.009	.014
Multivariable model 2^c^	1.02(0.99,1.04)	.130	.027	1.02(1.01,1.03)*	.043	.016	1.01(0.96,1.06)	.756	.043	0.95(0.91,0.99)*	.022	.013
Multivariable model 3^d^	1.01(0.99,1.03)	.252	.027	1.02(1.01,1.03)	.062	.014	1.00(0.95,1.05)	.898	.045	0.96(0.92,1.00)	.089	.016
**Low HDL-c**												
Cases = 2368												
Crude model	1.02(0.99,1.05)	.240	.027	1.02(1.00,1.04)**	.009	.016	1.03(0.97,1.09)	.381	.032	0.89(0.85,0.93)**	< .001	.013
Multivariable model 1^b^	1.02(0.99,1.05)	.071	.019	1.01(0.99,1.03)	.181	.029	1.04(0.98,1.10)	.144	.027	0.93(0.89,0.97)**	< .001	.011
Multivariable model 2^c^	1.02(1.00,1.04)	.080	.018	1.01(0.99,1.03)	.347	.034	1.03(0.97,1.09)	.338	.032	0.93(0.89,0.97)**	.001	.011
Multivariable model 3^d^	1.02(1.00,1.04)	.169	.025	1.00(0.98,1.02)	.632	.041	1.02(0.96,1.08)	.414	.032	0.94(0.90,0.98)**	.004	.011

Abbreviations: RR, Relative Risk; CI, confidence interval; PA, physical activity; HDL-c, high density lipoprotein-cholesterol.

^a^Light PA (<4.0 METs) includes leisurely stroll or walk. Moderate-vigorous PA (≥4.0 METs) includes faster walking, cross country walking, stair climbing, working in the garden, guided exercises and outdoor sports or at home or at the gym.

^b^Model 1, adjusted for age (continuous), sex, education level (illiterate /primary education, secondary education and academic/graduate), smoking status (never smoker, past smoker and current smoker), marital status (single/divorced, married and widower), familiar history of coronary heart disease (yes or no) and Mediterranean diet adherence (<8 or ≥9 items).

^c^Model 2, adjusted for variables in model 1 plus time spent in the other three self-reported activities. In addition, when models 1 and 2 were adjusted for obesity in type 2 diabetes, and for type 2 diabetes in obesity the associations remained unchanged.

^d^Model 3, adjusted for variables in model 2 plus the other four metabolic syndrome individuals components. All models were stratified by recruiting center.

^***¶***^ *P*-value * < .05, ** < .01.

*q*^^^ indicates false discovery rate-q value for multiple-testing using Benjamini-Hochberg test across the multiple associations between the exposure variables and the study outcomes.

Regarding the components of the MetS (Table 2), 1-h/day increase in TV-viewing was positively and independently associated with the prevalence of abdominal obesity (RR: 1.01, 95%CI: 1.00, 1.02) after adjustment for time spent in other activities and relevant covariates (model 2). Conversely, 1-h/day increase in MVPA was associated with an independent and significantly lower RR for abdominal obesity (RR: 0.97, 95%CI: 0.96, 0.98) and low-HDL-c (RR: 0.94, 95%CI: 0.90, 0.98). Further adjustment for each of the other four MetS components (model 3) had little impact on the risk estimates. No association between TV-viewing and MVPA, and the high blood pressure, hypertriglyceridemia and hyperglycemia components of the MetS were found in fully adjusted models. No association with the study outcomes was observed with sleeping and light intensity PA.

**Fig 1:**
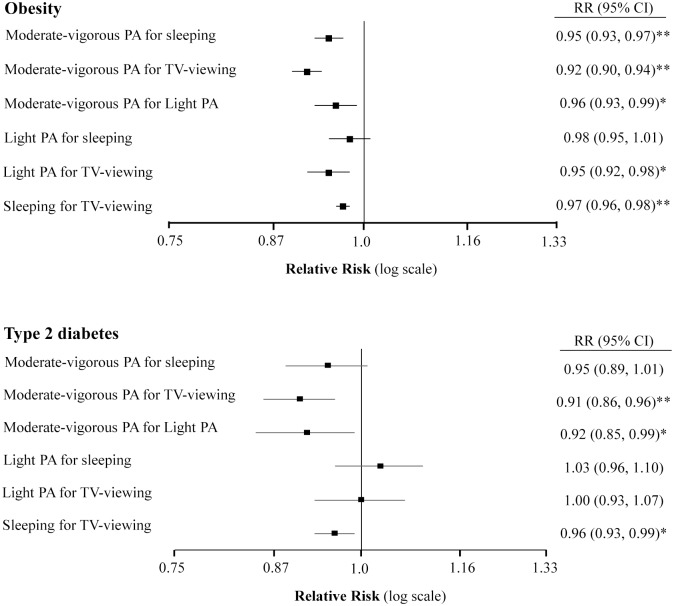
RR (95%CI) for obesity and diabetes to 60-min/day substitution among sleeping, TV-viewing and PA (n = 5776). Abbreviations: RR, Relative Risk; CI, confidence interval; PA, physical activity. Light PA (<4.0 METs) includes leisurely stroll or walk, Moderate-vigorous PA (≥4.0 METs) includes faster walking, cross country walking, stair climbing, working in the garden, guided exercises and outdoor sports or at home or at the gym. Multivariable model adjusted for age (continuous), sex, education level (illiterate/primary education, secondary education and academic/graduate), smoking status (never smoker, past smoker and current smoker), marital status (single/divorced, married and widower), familiar story of coronary hearth disease (yes or no) and Mediterranean diet adherence (<8 or ≥9 items). All models were stratified by recruiting center. *P*-value * < .05, ** < .01.

**Fig 2:**
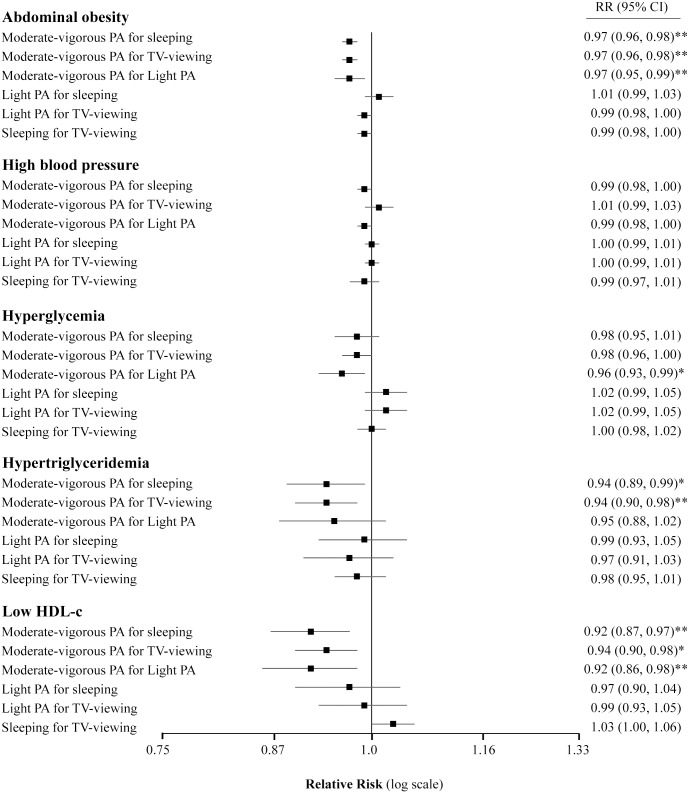
RR (95%CI) for metabolic syndrome components to 60-min/day substitution among sleeping, TV-viewing and PA (n = 5776). Abbreviations: RR, Relative Risk; CI, confidence interval; PA, physical activity. Light PA (<4.0 METs) includes leisurely stroll or walk. Moderate-vigorous PA (≥4.0 METs) includes faster walking, cross country walking, stair climbing, working in the garden, guided exercises and outdoor sports or at home or at the gym. Multivariable model adjusted for age (continuous), sex, education level (illiterate/primary education, secondary education and academic/graduate), smoking status (never smoker, past smoker and current smoker), marital status (single/divorced, married and widower), familiar story of coronary hearth disease (yes or no), Mediterranean diet adherence (<8 or ≥9 items) and the other four metabolic syndrome individuals components. All models were stratified by recruiting center. *P*-value * < .05, ** < .01.

Isotemporal substitution models are highlighted in Figs 1 and 2, and fully displayed in S1 Table. Theoretically substituting 1-h/day of MVPA for 1-h/day of sleeping, TV-viewing and light PA was associated with significantly lower prevalence of obesity (RR: 0.95, 95%CI: 0.93, 0.97; RR: 0.92, 95%CI: 0.90, 0.94 and RR: 0.96, 95%CI: 0.93, 0.99; respectively). Likewise, replacing 1-h/day of TV-viewing with 1-h/day of either light PA (RR: 0.95, 95%CI: 0.92, 0.98) or sleeping (RR: 0.97, 95%CI: 0.96, 0.98) was also inversely associated with obesity (Fig 1A). Similarly, substituting 1-h/day of MVPA for 1-h/day TV-viewing and light PA was associated with low prevalence of T2D (RR: 0.91, 95%CI: 0.86, 0.96; and RR: 0.92, 95%CI: 0.85, 0.99, respectively), as well as when substituting sleeping for TV-viewing (RR: 0.96, 95%CI: 0.93, 0.99) (Fig 1B).

Regarding components of the MetS, except for high blood pressure and hyperglycemia, the substitution of 1-h/day of MVPA for sleeping and TV-viewing was significantly associated with lower prevalence of abdominal obesity (RR:0.97; 95%CI:0.96, 0.98), hypertriglyceridemia (RR:0.94; 95%CI: 0.90, 0.98) and low HDL-c (RR 0.94; 95%CI: 0.90, 0.98). Only for abdominal obesity, hyperglycemia and low-HDL-c, substituting equal time of MVPA for light PA was associated to a lower risk (RR: 0.97, 95%CI: 0.95, 0.99; RR: 0.96, 95%CI: 0.93, 0.99; and RR: 0.92, 95%CI: 0.86, 0.98 respectively) (Fig 2). The opposite was also observed when MVPA was dropped out of the isotemporal substitution models (S1 Table). Replacing 1-h/day of other activities did not seem to be uniformly associated with the study outcomes, regardless of the activity type displaced (S1 Table).

**Fig 3:**
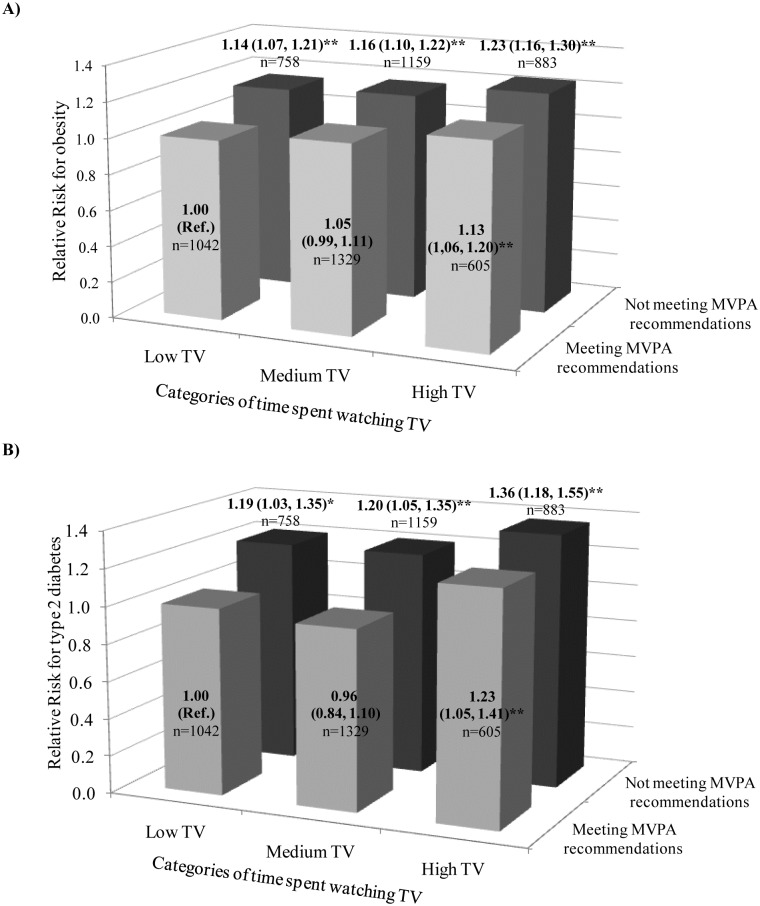
RR (95%CI) for obesity and diabetes for joint associations of TV-viewing and MVPA recommendations (n = 5776). Abbreviations: RR, Relative Risk; CI, confidence interval; MVPA, moderate-vigorous physical activity. Time spent in watching TV (in hours/day) was categorized as low TV (≤2h/day), medium TV (>2 to ≤4h/day) and high TV (>4h/day). Recommendations for MVPA according to the WHO 2010 (≥ 2.5h/wk). Moderate-vigorous PA (≥4.0 METs) includes faster walking, cross country walking, stair climbing, working in the garden, guided exercises and outdoor sports or at home or at the gym. Multivariable-adjusted model for age (continuous), sex, education level (illiterate/primary education, secondary education and academic/graduate), smoking status (never smoker, past smoker and current smoker), marital status (single/divorced, married and widower), familiar story of coronary hearth disease (yes or no), Mediterranean diet adherence (<8 or ≥9 items) and obesity and type 2 diabetes adjusted for each other. All models were stratified by recruiting center. *P*-value * < .05, ** *P* < .01.

Fig 3 shows joint associations by combining time spent in MVPA and TV-viewing on obesity and T2D prevalence. Fifty two percent of the participants met MVPA recommendations of ≥2.5 h/wk of which 20% belonged to high TV group, whereas this was true for 31.5% of the participants who did not meet MVPA recommendations. Compared to the reference group, those participants who did not meet the MVPA recommendations showed progressively higher RR for the prevalence of obesity parallel to time spent watching TV (RR 1.14 to 1.23, all *P* < .05). However, participants at the high TV group and not meeting MVPA recommendations had highest risks of being obese (RR: 1.23, 95%CI: 1.16, 1.30) (Fig 3A). Similar progressive pattern was observed concerning T2D risk. Those participants at the high TV group showed highest risks, regardless meeting (RR: 1.23, 95%CI: 1.05, 1.41) or not meeting (RR: 1.36, 95%CI: 1.18, 1.55) MVPA recommendations (Fig 3B), being the highest risk among the group of not meeting MVPA recommendation and high TV.

## Discussion

To the best of our knowledge, this is the first study that has examined the complex interrelationships between time spent in leisure-time PA, watching TV and sleep concerning to the prevalence of relevant cardiometabolic risk factors in senior adults with overweight or obesity and MetS at high CVD risk. The main findings in this report are that 1-h/day increase in MVPA was independently and significantly associated with lower prevalence of obesity, T2D and abdominal obesity and low HDL-c as independent components of the MetS. Contrary, detrimental associations were observed when increasing 1-h/day in TV-viewing for the same cardiometabolic risk factors, except for low HDL-c. Furthermore, when we theoretically replaced 1-h/day TV-viewing and sleeping by equal amounts of MVPA, beneficial effects were observed for these study outcomes, plus hypertriglyceridemia.

Our results on the independent associations between time spent in MVPA, TV-viewing and different cardiometabolic risk factors are in line with most of previous cross-sectional [13,20,21,27,42] and prospective studies [11,12,43,44] in different populations. For instance, a cross-sectional study from the large EPIC-Norfolk cohort of 14,189 adults aged ≥45 years showed that larger self-reported time spent in watching TV and in vigorous PA was positively and negatively associated, respectively, with CVD risk profile including markers of obesity, lipid profile and blood pressure [20]. Similar findings have been reported in other large cohorts regarding these outcomes [13,42] based on self-reported behaviors, and also using accelerometer-derived objective measurements for physical activity [25,27,45]. These previous results have been supported by various prospective studies, such as those from two large US cohorts in healthy middle-aged men [12] and women [11] showing positive associations between prolonged self-reported TV time and incidence of obesity and T2D. In contrast, increasing physical activity *per se* or moderate activities such as brisk walking were related to lower risk. In addition, there is a growing body of evidence from systematic reviews and meta-analyses of prospective and randomized controlled trials suggesting unfavorable links regarding TV-viewing or sedentary time, but favorable links with PA, in relation to obesity, T2D and cardiometabolic risk factors, such as lipids levels [2,3,8,46,47], as well as all-cause mortality [6], thereby supporting our study observations.

Although recently light PA has been suggested to play a positive role in preventing obesity, T2D and cardiometabolic risk factors, no strong associations have been found in our study. While there is some evidence for beneficial effects of light PA on health outcomes, such as blood pressure among physically inactive individuals with hypertension [48], it is not entirely unequivocal, particularly in regard to other cardiometabolic outcomes including body weight, body fat or lipid profile [48,49]. Therefore, it is plausible that the lack of associations observed in the literature and in our study might partly arise as a result of disparities in the design and selected populations, as well as the levels of light PA used to assess the effects on cardiometabolic health as they may be modest in comparison to those typically performed by adults [48]. Similarly, when looking at specific risk factors, we did not find any association between time spent in leisure-time PA or TV-viewing, and the prevalence of hyperglycemia or high blood pressure, which is in line with results from other authors [47,50], but not from all [13,45]. The lack of associations observed in our study concerning these outcomes may be due to the population studied, consisting of senior Mediterranean individuals at high CVD risk and using multiple medication. Taken together, further longitudinal studies are warranted to clarify these conflicting results in different populations.

It is recognized that sleeping >7–9 h/d is basic to promote optimal health [15,26], whereas outside this range sleep has been generally unfavorable linked with obesity [16,17], T2D and glucose metabolism disturbances [15,18], as well as other cardiometabolic risk factors [15]. Our study participants engaged an average of 7 sleeping hours per night on a regular basis and a large proportion (60%) of them reported sleeping between 7 to <9 h/day, hence potentially explaining the null association observed in our investigation.

Current public health strategies include separate messages advocating for increasing time in active behaviors [41] and reducing sedentary time [51]; our study found support for each of these. However, by employing theoretically isotemporal substitution modeling, a relatively less studied analytical approach, we further demonstrated that reallocating time from TV-viewing to additional MVPA was associated with a more favorable cardiometabolic profile. Based in our findings on obesity, T2D and some components of the MetS, it seems to exist different effect size in the associations between most activities and the study outcomes when assessing them independently than when applying isotemporal substitution modeling, even after adjusting for multiple potential confounders. For instance, we observed that 1-h/d of MVPA was independently associated to 6% protection against T2D, whereas when this activity replaced equal amounts of TV-viewing the protection increased up to 9% in our population of senior adults at higher risk; i.e. with overweight/obesity and MetS. In accordance to our results, recent reports applying same methodological approach and mostly using accelerometry have generally shown similar findings in middle-aged healthy persons [24–26] and adults with T2D [52]. Such studies have consistently evidenced that reallocating time from sedentary behaviors to equal amounts of either moderate PA, vigorous PA or MVPA was favorably associated to lower risk of obesity markers [25,26,52] as well as other CVD risk factors related to lipid profile and glucose metabolism [24–26], and even decreased risk of all cause-death [5]. Together with previous evidence, our findings support the notion that PA and sedentary behaviors have an individual impact on health outcomes, yet these effects may be underestimated if substitution effects among these activities are not considered. Given that PA and sedentary behaviors seem to strongly co-depend, it seems necessary to consider the isotemporal substitution methodology, as it provides with richer insights with relevant public health implications. Importantly, our investigation further addressed the combined associations of meeting/not-meeting MVPA recommendations and time spent watching TV in relation to obesity and T2D, indicating that the combination of not meeting MVPA recommendations and spending great time watching TV may be a strong risk factor for obesity. This message is of great importance given that evidence underpinning current international PA guidelines recommending ≥2.5 h/week of MVPA to reduce risk for obesity and T2D is limited to independent associations between PA and health outcomes [41]. In this context, our findings are in agreement with previous cross-sectional studies in healthy overweight adults showing highest obesity risks when low MVPA or insufficient PA levels were assessed in combination to greater TV time or sedentary behavior [27,53]. Furthermore, a recent 5-years prospective analysis on healthy overweight/obese adults revealed that larger MVPA time spent combined with lesser leisure time sitting was associated with lower risk of obesity [43].

Potential mechanisms may be driving the findings of our study. One such mechanism points out to the opposite contributions of PA and sedentary behaviors to energy balance by either promoting or hampering energy expenditure, respectively, while preserving the energy intake [54]. Additionally, it is plausible that time spent in watching TV results in increase total energy intake given that individuals tend to eat while watching TV—particularly high calorie, unhealthy foods [55]—which have been related to risk of obesity and diabetes [56]. Lastly, beneficial associations from PA are partly attributed to its ability to ameliorate body composition [57], improve glucose metabolism or enhance insulin sensitivity [3].

### Study limitations and strengths

Some methodological limitations should be acknowledged. First, the cross-sectional nature does not allow us to address causality and we admit the possibility of reverse causality bias as an alternative, non-causal, explanation of our results. Second, the present findings cannot be extrapolated to other population groups given that our study participants are senior adults with overweight/obesity and MetS. Additionally, although our study used self-reported PA and sedentary time which may be subject to potential biases, we used specifically validated questionnaires [33] and our results are according to those studies using accelerometry methods [24–27,45]. Furthermore, despite TV time has been suggested as a proxy for sedentary behaviors, the present study did not directly evaluate total sedentary time. Similarly, sleeping was limited to sleep duration in the present study, which does not account for the contribution of sleeping quality on cardiovascular health [58]. Finally, we acknowledge that the clinical significance provided by some relatively small increases in the risk prevalence of the study outcomes, remains unknown and deserves further exploration. Our study also has various strengths, including the large study sample of men and women, and the fully-adjusted analyses for potential confounding factors. Moreover, our study extensively addressed the complex interrelationships between different behaviors in relation to cardiometabolic risk factors by evaluating independent and combined associations, as well as by employing theoretically isotemporal substitution modeling.

## Conclusions

The results in our study with senior adults at high CVD risk, suggest that greater time spent on MVPA and fewer on sedentary behaviors is inversely associated with obesity, T2D and some of the components of the MetS. Public health strategies focusing on avoiding sedentary behaviors and substituting equivalent times for MVPA may represent a more appropriate approach in clinical practice for preventing cardiometabolic disorders, among aged populations at higher CVD risk. Further investigations should prospectively confirm our findings and elucidate potential mechanisms involved.

## Supporting information

S1 Text Additional list of the PREDIMED-PLUS trial. (PDF)

S1 Table (PDF)
