# Supplementary material for: Leisure-time physical activity, sedentary behaviors, sleep, and cardiometabolic risk factors at baseline in the PREDIMED-PLUS intervention trial: A cross-sectional analysis
Source: PLoS One. 2017 Mar 8;12(3):e0172253. doi: 10.1371/journal.pone.0172253 (PMC5342184; doi:10.1371/journal.pone.0172253)
Supplement: S1 Text — (PDF) [file pone.0172253.s001.pdf]

S1 Text

PREDIMED-PLUS Investigators:

Department of Preventive Medicine and Public Health, University of Navarra-Navarra Institute for Health Research and Primary Care Centres, Pamplona, Spain: Estefania Toledo, Miguel Ruiz-Canela, Cristina Razquin, Maira Bes-Rastrollo, Ana Sanchez Tainta, Beatriz SanJulian Aranguren, Estibaliz Goñi, Irene Barrientos, Maria Canales, Anais Rico, Javier Basterra Gortari, Ana Garcia Arellano, Javier Diez-Espino, Oscar Lecea Juarez, Juan Carlos Cenoz Osinaga, Javier Bartolome Resano, Ana Sola-Larraza, Elisa Lozano-Oloriz, Begoña Cano-Valles, Sonia Eguaras, Elena Pascual Roquet-Jalmar, Iñigo Galilea-Zabalza, Hana Lancova, Raul Ramallal, Mª Luisa Garcia-Perez, Vicente Estremera-Urabayen, Mª Jose Ariz-Arnedo, Carmen Hijos-Larraz, Cristina Fernandez Alfaro, Begoña Iñigo-Martinez, Ramon Villanueva Moreno, Sonia Martin-Almendros, Luisa Barandiaran-Bengoetxea, Carmen Fuertes-Goñi, Ana Lezaun-Indurain, Mª Jose Guruchaga-Arcelus, Oscar Olmedo-Cruz, Begoña Iñigo-Martínez, Luis Escriche-Erviti, Roberto Ansorena-Ros, Rosario Sanmatin Zabaleta, Jon Apalategi-Lasa, Jerusalen Villanueva-Telleria, Mª Mar Hernández-Espinosa, Isabel Arroyo-Bergera, Lissy Herrera-Valdez, Lourdes Dorronsoro-Dorronsoro

University Rovira i Virgili, Reus, Spain: Roser Pedret Llaberia, Rosi Gonzalez, Ramon Sagarra Álamo, Francesc París Palleja, Josep Balsells, Josep Mª Roca, Teresa Basora Gallisa, Jesus Vizcaino, Pilar Llobet Alpizarte, Carme Anguera Perpiñá, Montse Llauradó Vernet, Clara Caballero, Montserrat Garcia Barco, Mª D. Morán Martínez, Joaquin García Rosselló, Albert Del Pozo, Carmina Poblet Calaf, Pierre Arcelin Zabal, Xavier Floresví, Marta Ciutat Benet, Antoni Palau Galindo, Joan Josep Cabré Vila, Fernando Dolz Andrés, Juan Boj Casajuana, Meritxell Ricard, Francisco Saiz, Anna Isach, Mar Sanchez Marin Martinez, Mónica Bulló, Nancy Babio, Nerea Becerra-Tomás, Cintia Ferreira-Pêgo, Glòria Mestres, Josep Basora, Guillermo Mena-Sánchez, Laura Barrubés Piñol, Marta Gil Segura, Christopher Papandreou

Universidad de Valencia, Universidad Jaume I and Conselleria de Sanidad de la Generalitat Valenciana, Valencia, Spain: González JI, Sorlí JV, Portolés O, Sáiz C, Ortega-Azorín C, Barragán R, Carrasco P, Asensio EM, Fernández-Carrión R, Férriz E, González-Monje I, Guillém-Sáiz P, Quiles L, Coltell O, Carratalá-Calvo A, Valero-Barceló C, Osma R, Antón F, Mir C, Sánchez-Navarro S, González-Gallego I, Bort-Llorca L, Pérez-Ollero L, Giner-Valero M, Nadal-Sayol J, Pascual V, Martínez-Pérez M, Riera C, Belda MV, Medina A, Miralles E, Ramírez-Esplugues MJ, Rojo M

Servicio de Endocrinología, Hospital del Mar, Barcelona. Departament de Medicina, Universitat Autònoma de Barcelona, Barcelona, Spain: Montserrat Fitó, Olga Castañer, Miguel Ángel Muñoz, Maria Dolors Zomeño, Laura Torres, Mireia Quifer, Regina Llimona, Roberto Elosua, Jaume Marrugat, Joan Vila, Juan Jose Chillaron Jordan, Juana Antonia Flores Lerroux, David Benaiges Boix, Daniel Muñoz-Aguayo, Susanna Tello, Marta Cabañero, Leny Franco, Helmut Schröder, Rafael de la Torre, Casimira Medrano, Joan Bayó, Maria Teresa García, Vanesa Robledo, Pilar Babi, Erik

Canals, Núria Soldevila, Lourdes Carrés, Carme Roca, Maria de la Serra Comas, Griselda Gasulla, Xavier Herraiz, Alba Martínez

Department of Nutrition, Food Sciences, and Physiology, Center for Nutrition Research, University of Navarra, Pamplona, Spain: Itziar Abete, Irene Cantero, Carmen Cristobo, Idoia Ibero-Baraibar, Marian Zulet, Javier Ágreda Martínez, Mª Dolores Lezáun Burgui, Nuria Goñi Ruiz, Rafael Bartolomé Resano, Eugenia Cano Cáceres, Teresa Elcarte López, Elena Echarte Osacain, Beatriz Pérez Sanz, Itziar Blanco Platero, Simón Antonio Andueza Azcárate, Álvaro Gimeno. Aznar, Eugenia Ursúa Sesma, Benito Ojeda Bilbao, Javier Martinez Jarauta, Lourdes Ugalde Sarasa, Blanca Rípodas Echarte, Mª Victoria Güeto Rubio

Hospital Son Espases (HUSE) and Instituto de Investigación Sanitaria de Palma (IdISPa), Palma de Mallorca, Spain: Miquel Fiol, Manuel Moñino, Antoni Colom, Margarita Morey, Rocío Zamanillo, Aina Yáñez, Jadwiga Konieczna, Joan Llobera, Joana Ripoll, Rafael Prieto, Felix Grases, Antonia Costa, Susana Munuera, Francisco Tomás, Francisca Fiol, Antoni Jover, Juana Mª Janer, Catalina Vallespir, Isabel Mattei, Natalia Feuerbach, Maria del Mar Sureda, Silvia Vega, Lourdes Quintana, Aina Fiol, Miriam Amador, Susana González

University of Miguel Hernández, Alicante, Spain: Eva M. Navarrete Muñoz, Sandra González Palacios, Laura Torres Collado, Desirée Valera Gran, Manuela García de la Hera, Laura Compañ Gabucio, Alejandro Oncina Canovas, Orozco Beltran, Domingo, Ascensión Luisa Santos Hernández, Blas Cloquell Rodrigo, María Consuelo Altozano Rodado, María Vicenta Hernándis Marsán, Salvador Pertusa Martínez, Alberto Asensio, Norma Iranzo García, Inma Candela García, Noelia Fernándis Brufal, Mª Carmen Martínez Vergara, Josefa Román Maciá, Mª Teresa Cano Sánchez, Juan Manuel Zazo, Salvador Juan Miralles Gisbert, Andrés González Botella, Carmen M. López García, Rafael Valls Enguix, Cristina Gisbert Sellés

University Hospital of Alava, Vitoria, Spain: Alfonso Casi Casanellas, María Luz Arnal Otero, Jesús Ortueta Martínez De Arbulo, Julia Vinagre Morgado, Jorge Romeo Ollora, Javier Urraca, María Isabel Sarriegui Carrera, Francisco Javier Toribio, Elena Magán, Alfonso Rodríguez, Sagrario Castro Madrid, Maria Teresa Gómez Merino, Manuela Rodríguez Jiménez, Maxi Gutiérrez Jodra, Begoña López Alonso, Jesús Iturralde Iriso, Concepción Pascual Romero, Ana Izquierdo De La Guerra, Sara Roiz Ortiz

Reina Sofia University Hospital. IMIBIC. University of Córdoba, Córdoba, Spain: Jose Lopez-Miranda, Francisco Perez-Jimenez, Javier Delgado-Lista, Pablo Perez-Martinez, Francisco Fuentes-Jimenez, Juan Francisco Alcala-Diaz, Francisco Gomez-Delgado, Ana I. Perez-Caballero, Ana Leon Acuña, Jose David Torres-Peña, Juan Criado-Garcia, Ana I. Jimenez-Morales, Fernando Rodriguez-Cantalejo, Javier Caballero-Villarraso, Gracia Quintana-Navarro, Patricia Peña Orihuela, Antonio Camargo Garcia, Carmen Marin Hinojosa, Ana Ortiz Morales, Vanesa Navarro, Elena Yubero Serrano

Virgen de la Victoria Hospital, Málaga University, Málaga, Spain: Francisco J Tinahones, M Rosa Bernal López, Manuel Macías González, Josefina Ruiz Nava, Nieves

del Rocío Casal Nievas, José Carlos Fernández García, Araceli Muñoz Garach, Alberto Vilches Pérez, Arantxa González Banderas, Juan Alcaide Torres, Antonio Vargas Candela, María León Fernández, Rosa Hernández Robles, Sonia Santamaría Fernández, José Manuel Marín, Sergio Valdés Hernández, Juan Carlos Villalobos, Antonio Ortiz.

Department of Internal Medicine, Hospital Clínic, IDIBAPS August Pi i Sunyer Biomedical Research Institute, University of Barcelona, Barcelona, Spain: M. Domenech, C. Sierra, M. Camafort, Gabriel Fontana, S. Castro, M. Sadurni, C. Viñas, P. Villanueva, R. Soriano, Rosa Casas, M. de la Poza, J.M. Cots, J. M. Llovet, C. Carbonell, Y. García, J. Altirriba, V. Aragunde

University of Málaga, Málaga, Spain: Enrique Gómez-Gracia, Julia Warnberg, Jessica Pérez-López, Juan Carlos Benavente, Francisco Javier Barón-López, José Ramón Alvero-Cruz, Maria del Carmen Rodríguez-Martínez, Eugenio Contreras, Francisco Jesús Carmona, Rosa Carabaño, Salvador Torres, María Isabel Alcalá, Francisco Javier Vázquez, Carolina Gallego, Anabel Jiménez, Luna Begines, Edelys Crespo, Paula Sánchez

Department of Family Medicine, Research Unit, Primary Care Division, Sevilla, Spain: José Manuel Santos-Lozano, Manuel Ortega-Calvo, Francisco José García-Corte, Leticia Miró-Moriano, Cristina Domínguez-Espinaco, Lidia Mellado-Martín, Sergio Vaquero-Díaz, Inmaculada Rivera-Benítez, Pablo Iglesias-Bonilla, Pilar Román-Torres, Eduardo Mayoral-Sánchez, María Caballero-Valderrama, Amparo Santos-Calonge, Carmen Toro-Cortés, Yolanda Corchado-Albalat, Carmen Lama-Herrera, Víctor Urbano-Fernández

IUIBS, University of Las Palmas de Gran Canaria, Las Palmas, Spain: J Álvarez-Pérez, LT Casañas-Quintana, J Pérez-Cabrera, F Díaz-Collado, C Ruano-Rodríguez, A Sánchez-Villegas, RB García-Guerra, JB Álvarez-Álvarez, F Sarmiento-De La Fe, CD Méndez-García, LT García-Pastor, C Simon-García, BV Díaz-González, JM Castillo-Anzalas, RE Sosa-Also, J Medina-Ponce, MI Travesí-García

Bellvitge Biomedical Research Institute (IDIBELL), Hospital Universitari de Bellvitge, Hospitalet de Llobregat, Barcelona, Spain: Xavier Pintó, Emili Corbella, Ana Galera, Elsa de la Cruz, Ferran Trias, Marta Fanlo, Rosaura Figueras, Xavier Corbella, M. Ángeles Rodríguez, Ramon Pujol, Pilar Martín, Jordi Vilaseca, Hannia Lafuente, Rosa Freixedas, Alicia Val

University of the Balearic Islands, Palma de Mallorca, Spain: Alicia Julibert, Emma Argelich, Caterina Terrassa, Cristina Bouzas, Laura Gallardo, Manuela Abbate, Lucía Ugarriza Tomás Rodriguez, Tomás Ripoll, Escarlata Angullo, Belén García

Departament of Preventive Medicine and Public Health, University of Granada, Granada, Spain: Eva Mª Garrido Garrido, Ana Baena Dominguez, Francisco García Jiménez, Esther Thomas Carazo, Antonio Jesús Turnes González, Francisco González Jiménez, Francisco Padilla Ruiz, Javier Machado Santiago, Jose F. Guillén Solvas, Armando Pueyos Sánchez

Department of Endocrinology and Nutrition, Hospital Clínic, Barcelona, Spain: Carla Mestre Reina, Emilio Ortega Martínez de la Victoria, Irene Vinagre Torres, Judit Viaplana Masclans, José Ignacio Pla Puig, Tania-Marisa, Freitas-Simoes

University of Jaen, Jaen, Spain: Jose Juan Gaforio, Sandra Moraleda, Norberto Liétor, José Ignacio Peis, Tomás Ureña, Montserrat Rueda

Grupo de Investigación en Interacciones Gen-Ambiente y Salud, Universidad de León y Gerencia de Atención Primaria. Sacyl, León, Spain: Vicente Martín, Serafín de Abajo Olea, Miguel Escobar Fernández, Jose Pedro Fernández Vázquez, Jaime López de la Iglesia, Juan Ignacio López Gil, Elena Carriedo Ule, Manuel Rodríguez Bul, Abdurrahman Adlbi Sibai, Alberto García Hernández

Department of Cardiovascular Epidemiology and Population Genetics, Centro Nacional de Investigaciones Cardiovasculares and Madrid Institute for Advanced Studies (IMDEA) Food Institute, Madrid, Spain: Lidia Daimiel, José Mª Ordovás, Víctor Micó Moreno, Mª José Concejo Carranza, Javier Muñoz Gutiérrez, Mercedes Adrián Sanz, Yolanda de la Fuente Cortés

Department of Endocrinology and Nutrition, University Hospital Fundación Jiménez Díaz, Madrid, Spain: Ana Isabel de Cos Blanco, Ana Prieto Moreno, Angela Candelas, Sebastian Mas Fontao, Sonsoles Gutierrez, Roberto Dominguez, Miguel Aganzo

Hospital Clínico San Carlos. IDISSC, Madrid, Spain: Macarena Torrego Ellacuría, Pilar Matía Martín, Carmen Moreno Lopera, Ana Barabash Bustelo, María Ceballos de Diego, Concepción Aragoneses Isabel
