# Supplementary material for: Leisure-time physical activity, sedentary behaviors, sleep, and cardiometabolic risk factors at baseline in the PREDIMED-PLUS intervention trial: A cross-sectional analysis
Source: PLoS One. 2017 Mar 8;12(3):e0172253. doi: 10.1371/journal.pone.0172253 (PMC5342184; doi:10.1371/journal.pone.0172253)
Supplement: S1 Table — (PDF) [file pone.0172253.s002.pdf]

S1 Table 1. Multivariable-adjusted RR (95% CI) for obesity[a] per 60-min/day substitution in sleeping, watching TV and PA

**Substitution Model** (Substitution of 60 min/day of an activity to replace 60 min/day for one of the remaining activities)	**Sleeping**	**TV-watching**	**Light PA[b]**	**Moderate-vigorous PA[b]**	**Total discretionary time[c]**
**Substitution model A : Sleeping is dropped**					
Crude model	Dropped	1.03 (1.02, 1.04)**	0.98 (0.95, 1.01)	0.95 (0.92, 0.98)**	1.00 (0.99, 1.01)
Multivariable model[d]	Dropped	1.03 (1.01, 1.05)**	0.98 (0.95, 1.01)	0.95 (0.93, 0.97)**	1.00 (0.99, 1.01)
**Substitution model B : TV-Watching is dropped**					
Crude model	0.97 (0.96, 0.98)**	Dropped	0.95 (0.92, 0.98)**	0.92 (0.90, 0.94)**	1.02 (1.01, 1.03)**
Multivariable model[d]	0.97 (0.96, 0.98)**	Dropped	0.95 (0.92, 0.98)*	0.92 (0.90, 0.94)**	1.03 (1.02, 1.04)**
**Substitution model C : Light PA is dropped**					
Crude model	1.02 (0.99, 1.05)	1.05 (1.02, 1.08)**	Dropped	0.97 (0.93, 1.01)	0.98 (0.95, 1.01)
Multivariable model[d]	1.02 (0.99, 1.05)	1.05 (1.02, 1.08)**	Dropped	0.96 (0.93, 0.99)*	0.98 (0.95, 1.01)
**Substitution model D: Moderate-vigorous PA is dropped**					
Crude model	1.05 (1.02, 1.08)**	1.09 (1.06, 1.12)**	1.03 (0.99, 1.07)	Dropped	0.95 (0.93, 0.97)**
Multivariable model[d]	1.05 (1.02, 1.08)**	1.08 (1.06, 1.10)**	1.03 (0.99, 1.07)	Dropped	0.95 (0.93, 0.97)**

Abbreviations: CI, confidence interval; RR, Relative Risk; PA, physical activity. [a]Obesity is defined as BMI ≥30 kg/m^2. [b]Light PA (<4.0 METs) includes leisurely stroll or walk. Moderate-vigorous PA (≥4.0 METs) includes faster walking, cross country walking, stair climbing, working in the garden, guided exercises and outdoor sports or at home or at the gym. [c]Total discretionary time= sum of time spent in sleeping, TV-watching, light PA and moderate-vigorous PA. The coefficient for total discretionary time represents the omitted activity component (e.g. sleeping for substitution model A, TV-watching for substitution model B and so on). [d]Multivariable-adjusted model for age (continuous), gender, education level (illiterate/primary education, secondary education and academic/graduate), smoking status (never smoker, past smoker, current smoker), marital status (single/divorced, married and widower), familiar history of coronary heart disease (yes or no) and Mediterranean diet adherence (<8 or ≥9 items). All models were stratified by recruiting center. * P<0.05, ** P<0.01. Additionally, the models were adjusted for type 2 diabetes and the Relative Risk remained unchanged.

S1 Table 2. Multivariable-adjusted RR (95% CI) for diabetes per 60-min/day substitution in sleeping, watching TV and PA

**Substitution Model** (Substitution of 60 min/day of an activity to replace 60 min/day for one of the remaining activities)	**Sleeping**	**TV-watching**	**Light PA[a]**	**Moderate-vigorous PA[a]**	**Total discretionary time[b]**
**Substitution model A : Sleeping is dropped**					
Crude model	Dropped	1.05 (1.01, 1.09)*	1.05 (0.97, 1.13)	0.98 (0.93, 1.03)	1.00 (0.97, 1.03)
Multivariable model[c]	Dropped	1.05 (1.01, 1.09)*	1.03 (0.96, 1.10)	0.95 (0.89, 1.01)	1.00 (0.96, 1.04)
**Substitution model B : TV-Watching is dropped**					
Crude model	0.95 (0.92, 0.98)*	Dropped	1.01 (0.94, 1.08)	0.94 (0.90, 0.98)**	1.05 (1.03, 1.07)**
Multivariable model[c]	0.96 (0.93, 0.99)*	Dropped	1.00 (0.93, 1.07)	0.91 (0.86, 0.96)**	1.04 (1.02, 1.06)**
**Substitution model C : Light PA is dropped**					
Crude model	0.95 (0.88, 1.02)	0.99 (0.92, 1.06)	Dropped	0.94 (0.86, 1.02)	1.06 (0.99, 1.13)
Multivariable model[c]	0.97 (0.90, 1.04)	1.00 (0.93, 1.07)	Dropped	0.92 (0.85, 0.99)*	1.05 (0.98, 1.12)
**Substitution model D: Moderate-vigorous PA is dropped**					
Crude model	1.02 (0.96, 1.08)	1.06 (1.01, 1.11)*	1.06 (0.98, 1.14)	Dropped	0.98 (0.93, 1.03)
Multivariable model[c]	1.06 (1.00, 1.12)	1.11 (1.05, 1.17)**	1.10 (1.02, 1.18)**	Dropped	0.94 (0.89, 0.99)*

Abbreviations: CI, confidence interval; RR, Relative Risk; PA, physical activity. [a]Light PA (<4.0 METs) includes leisurely stroll or walk. Moderate-vigorous PA (≥4.0 METs) includes faster walking, cross country walking, stair climbing, working in the garden, guided exercises and outdoor sports or at home or at the gym. [b]Total discretionary time= sum of time spent in sleeping, TV-watching, light PA and moderate-vigorous PA. The coefficient for total discretionary time represents the omitted activity component (e.g. sleeping for substitution model A, TV-watching for substitution model B and so on). [c]Multivariable-adjusted model for age (continuous), gender, education level (illiterate/primary education, secondary education and academic/graduate), smoking status (never smoker, past smoker, current smoker), marital status (single/divorced, married and widower), familiar history of coronary heart disease (yes or no) and Mediterranean diet adherence (<8 or ≥9 items). All models were stratified by recruiting center. * P<0.05, ** P<0.01. Additionally, the models were adjusted for obesity and the Relative Risk remained unchanged.

S1 Table 3. Multivariable-adjusted RR (95% CI) for abdominal obesity[a] per 60-min/day substitution in sleeping, watching TV and PA

**Substitution Model** (Substitution of 60 min/day of an activity to replace 60 min/day for one of the remaining activities)	**Sleeping**	**TV-watching**	**Light PA[b]**	**Moderate-vigorous PA[b]**	**Total discretionary time[c]**
**Substitution model A : Sleeping is dropped**					
Crude model	Dropped	1.01 (1.00, 1.02)*	1.00 (0.99, 1.01)	0.96 (0.95, 0.97)**	1.00 (0.99, 1.01)
Multivariable model 1[d]	Dropped	1.00 (0.99, 1.01)	1.00 (0.98, 1.02)	0.97 (0.96, 0.98)**	1.00 (0.99, 1.01)
Multivariable model 2[e]	Dropped	1.01 (0.99, 1.03)	1.01 (0.99, 1.03)	0.97 (0.96, 0.98)**	1.01 (0.99, 1.01)
**Substitution model B : TV-Watching is dropped**					
Crude model	0.99 (0.98, 1.00)	Dropped	0.99 (0.98, 1.00)	0.95 (0.94, 0.96)**	1.01 (1.02, 1.03)**
Multivariable model 1[d]	0.99 (0.98, 1.00)	Dropped	0.99 (0.97, 1.01)	0.96 (0.95, 0.97)**	1.01 (1.00, 1.02)**
Multivariable model 2[e]	0.99 (0.98, 1.00)	Dropped	0.99 (0.98, 1.00)	0.97 (0.96, 0.98)**	1.01 (1.00, 1.02)**
**Substitution model C : Light PA is dropped**					
Crude model	0.99 (0.98, 1.00)	1.01 (1.00, 1.02)	Dropped	0.96 (0.95, 0.97)**	1.00 (0.99, 1.01)
Multivariable model 1[d]	0.99 (0.98, 1.00)	1.00 (0.99, 1.01)	Dropped	0.97 (0.95, 0.99)**	1.00 (0.99, 1.02)
Multivariable model 2[e]	0.99 (0.98, 1.00)	1.00 (0.99, 1.01)	Dropped	0.97 (0.95, 0.99)**	1.00 (0.99, 1.02)
**Substitution model D: Moderate-vigorous PA is dropped**					
Crude model	1.04 (1.02, 1.06)**	1.05 (1.04, 1.06)**	1.04 (1.02, 1.06)**	Dropped	0.96 (0.95, 0.97)**
Multivariable model 1[d]	1.03 (1.02, 1.05)**	1.04 (1.02, 1.05)**	1.03 (1.02, 1.05)**	Dropped	0.97 (0.96, 0.98)**
Multivariable model 2[e]	1.03 (1.02, 1.05)**	1.04 (1.02, 1.05)**	1.03 (1.02, 1.05)**	Dropped	0.97 (0.96, 0.98)**

Abbreviations: CI, confidence interval; RR, Relative Risk; PA, physical activity. [a]Abdominal obesity is defined as waist circumference ≥88 cm in women or ≥102 cm in men. [b]Light PA (<4.0 METs) includes leisurely stroll or walk. Moderate-vigorous PA (≥4.0 METs) includes faster walking, cross country walking, stair climbing, working in the garden, guided exercises and outdoor sports or at home or at the gym. [c]Total discretionary time= sum of time spent in sleeping, TV-watching, light PA and moderate-vigorous PA. The coefficient for total discretionary time represents the omitted activity component (e.g. sleeping for substitution model A, TV-watching for substitution model B and so on). [d]Multivariable-adjusted model for age (continuous), gender, education level (illiterate/primary education, secondary education and academic/graduate), smoking status (never smoker, past smoker and current smoker), marital status (single/divorced, married and widower), familiar history of coronary heart disease (yes or no) and Mediterranean diet adherence (<8 or ≥9 items). [e]Multivariable-adjusted model for variables in model 1 plus the other four metabolic syndrome individuals components. All models were stratified by recruiting center. *P<0.05, ** P<0.01.

S1 Table 4. Multivariable-adjusted RR (95% CI) for high blood pressure[a] per 60-min/day substitution in sleeping, watching TV and PA

**Substitution Model** (Substitution of 60 min/day of an activity to replace 60 min/day for one of the remaining activities)	**Sleeping**	**TV-watching**	**Light PA[b]**	**Moderate-vigorous PA[b]**	**Total discretionary time[c]**
**Substitution model A : Sleeping is dropped**					
Crude model	Dropped	1.00 (0.99, 1.01)	1.01 (0.99, 1.03)	0.99 (0.98, 1.00)	1.00 (0.99, 1.01)
Multivariable model 1[d]	Dropped	1.00 (0.99, 1.01)	1.00 (0.99, 1.01)	0.99 (0.97, 1.01)	1.00 (0.99, 1.01)
Multivariable model 2[e]	Dropped	1.00 (0.99, 1.01)	1.00 (0.99, 1.01)	0.99 (0.98, 1.00)	1.00 (0.99, 1.01)
**Substitution model B : TV-Watching is dropped**					
Crude model	0.99 (0.98, 1.00)	Dropped	1.00 (0.99, 1.01)	1.03 (1.01, 1.05)*	1.01 (1.00, 1.02)
Multivariable model 1[d]	0.99 (0.98, 1.00)	Dropped	1.00 (0.99, 1.01)	1.01 (0.99, 1.03)	1.01 (1.00, 1.02)
Multivariable model 2[e]	0.99 (0.97, 1.01)	Dropped	1.00 (0.99, 1.01)	1.01 (0.99, 1.03)	1.01 (1.00, 1.02)
**Substitution model C : Light PA is dropped**					
Crude model	0.99 (0.97, 1.01)	0.99 (0.98, 1.00)	Dropped	1.00 (0.99, 1.01)	1.00 (0.99, 1.01)
Multivariable model 1[d]	0.99 (0.97, 1.01)	1.00 (0.98, 1.02)	Dropped	0.99 (0.98, 1.00)	1.00 (0.99, 1.01)
Multivariable model 2[e]	0.99 (0.98, 1.00)	1.00 (0.99, 1.01)	Dropped	0.99 (0.98, 1.00)	1.01 (1.00, 1.02)
**Substitution model D: Moderate-vigorous PA is dropped**					
Crude model	1.00 (0.99, 1.01)	0.97 (0.96, 0.98)**	1.01 (1.00, 1.02)	Dropped	1.03(1.01, 1.05)**
Multivariable model 1[d]	1.01 (0.99, 1.02)	0.99 (0.96, 1.02)	1.01 (0.99, 1.03)	Dropped	1.00 (0.99, 1.01)
Multivariable model 2[e]	1.00 (0.98, 1.02)	0.99 (0.97, 1.01)	1.01 (1.00, 1.02)	Dropped	0.99 (0.98, 1.00)

Abbreviations: CI, confidence interval; RR, Relative Risk; PA, physical activity. [a]High blood pressure is defined as systolic and/or diastolic blood pressure ≥130/85 mmHg or use of antihypertensive medication. [b]Light PA (<4.0 METs) includes leisurely stroll or walk. Moderate-vigorous PA (≥4.0 METs) includes faster walking, cross country walking, stair climbing, working in the garden, guided exercises and outdoor sports or at home or at the gym. [c]Total discretionary time= sum of time spent in sleeping, TV-watching, light PA and moderate-vigorous PA. The coefficient for total discretionary time represents the omitted activity component (e.g. sleeping for substitution model A, TV-watching for substitution model B and so on). [d]Multivariable-adjusted model for age (continuous), gender, education level (illiterate/primary education, secondary education and academic/graduate), smoking status (never smoker, past smoker and current smoker), marital status (single/divorced, married and widower), familiar history of coronary heart disease (yes or no) and Mediterranean diet adherence (<8 or ≥9 items). [e]Multivariable-adjusted model for variables in model 1 plus the other four metabolic syndrome individuals components. All models were stratified by recruiting center. * P<0.05, ** P<0.01..

S1 Table 5. Multivariable-adjusted RR (95% CI) for hyperglycemia[a] per 60-min/day substitution in sleeping, watching TV and PA

**Substitution Model** (Substitution of 60 min/day of an activity to replace 60 min/day for one of the remaining activities)	**Sleeping**	**TV-watching**	**Light PA[b]**	**Moderate-vigorous PA[b]**	**Total discretionary time[c]**
**Substitution model A : Sleeping is dropped**					
Crude model	Dropped	1.00 (0.98, 1.02)	1.03 (1.00, 1.06)	0.99 (0.96, 1.02)	1.01 (1.00, 1.02)
Multivariable model 1[d]	Dropped	1.00 (0.98, 1.02)	1.02 (0.99, 1.05)	0.98 (0.95, 1.01)	1.01 (1.00, 1.02)
Multivariable model 2[e]	Dropped	1.00 (0.98, 1.02)	1.02 (0.99, 1.05)	0.98 (0.95, 1.01)	1.01 (1.00, 1.02)
**Substitution model B : TV-Watching is dropped**					
Crude model	0.99 (0.98, 1.01)	Dropped	1.02 (0.99, 1.05)	0.99 (0.97, 1.01)	1.01 (1.00, 1.02)
Multivariable model 1[d]	1.00 (0.98, 1.02)	Dropped	1.02 (0.99, 1.05)	0.98 (0.96, 1.00)	1.00 (0.99, 1.01)
Multivariable model 2[e]	1.00 (0.98, 1.02)	Dropped	1.02 (0.99, 1.05)	0.98 (0.96, 1.00)	1.01 (1.00, 1.02)
**Substitution model C : Light PA is dropped**					
Crude model	0.97 (0.94, 1.00)	0.98 (0.95, 1.01)	Dropped	0.96 (0.93, 0.99)*	1.03 (1.01, 1.05)*
Multivariable model 1[d]	0.98 (0.95, 1.01)	0.98 (0.95, 1.01)	Dropped	0.96 (0.93, 0.99)*	1.03 (1.00, 1.06)
Multivariable model 2[e]	0.98 (0.95, 1.01)	0.98 (0.95, 1.01)	Dropped	0.96 (0.93, 0.99)*	1.03 (1.00, 1.06)
**Substitution model D: Moderate-vigorous PA is dropped**					
Crude model	1.01 (0.98, 1.04)	1.01 (0.98, 1.04)	1.03 (1.01, 1.05)*	Dropped	1.00 (0.98, 1.02)
Multivariable model 1[d]	1.02 (0.99, 1.05)	1.02 (0.99, 1.05)	1.04 (1.01, 1.07)*	Dropped	0.98 (0.96, 1.00)
Multivariable model 2[e]	1.02 (0.99, 1.05)	1.02 (0.99, 1.05)	1.04 (1.01, 1.07)*	Dropped	0.98 (0.96, 1.00)

Abbreviations: CI, confidence interval; RR, Relative Risk; PA, physical activity. [a]Hyperglycemia is defined as fasting plasma glucose ≥5.5 mmol/L or ≥100 mg/dL) or the use of medication for elevated glucose levels. [b]Light PA (<4.0 METs) includes leisurely stroll or walk. Moderate-vigorous PA (≥4.0 METs) includes faster walking, cross country walking, stair climbing, working in the garden, guided exercises and outdoor sports or at home or at the gym. [c]Total discretionary time= sum of time spent in sleeping, TV-watching, light PA and moderate-vigorous PA. The coefficient for total discretionary time represents the omitted activity component (e.g. sleeping for substitution model A, TV-watching for substitution model B and so on). [d]Multivariable-adjusted model for age (continuous), gender, education level (illiterate/primary education, secondary education and academic/graduate), smoking status (never smoker, past smoker and current smoker), marital status (single/divorced, married and widower), familiar history of coronary heart disease (yes or no) and Mediterranean diet adherence (<8 or ≥9 items). [e]Multivariable-adjusted model for variables in model 1 plus the other four metabolic syndrome individuals components. All models were stratified by recruiting center. *P<0.05, ** P<0.01.

S1 Table 6. Multivariable-adjusted RR (95% CI) for hypertriglyceridemia[a] per 60-min/day substitution in sleeping, watching TV and PA

**Substitution Model** (Substitution of 60 min/day of an activity to replace 60 min/day for one of the remaining activities)	**Sleeping**	**TV-watching**	**Light PA[b]**	**Moderate-vigorous PA[b]**	**Total discretionary time[c]**
**Substitution model A : Sleeping is dropped**					
Crude model	Dropped	1.00 (0.97, 1.03)	0.97 (0.91, 1.03)	0.92 (0.87, 0.97)**	1.02 (1.00, 1.04)
Multivariable model 1[d]	Dropped	1.01 (0.98, 1.04)	0.98 (0.92, 1.04)	0.93 (0.88, 0.98)**	1.02 (0.99, 1.05)
Multivariable model 2[e]	Dropped	1.02 (0.98, 1.06)	0.99 (0.93, 1.05)	0.94 (0.89, 0.99)*	1.01 (0.98, 1.04)
**Substitution model B : TV-Watching is dropped**					
Crude model	0.99 (0.96, 1.01)	Dropped	0.97 (0.91, 1.03)	0.92 (0.88, 0.96)**	1.01 (0.99, 1.03)
Multivariable model 1[d]	0.98 (0.95, 1.01)	Dropped	0.97 (0.91, 1.03)	0.92 (0.88, 0.96)**	1.03 (1.01, 1.05)**
Multivariable model 2[e]	0.98 (0.95, 1.01)	Dropped	0.97 (0.91, 1.03)	0.94 (0.90, 0.98)**	1.03 (1.01, 1.05)*
**Substitution model C : Light PA is dropped**					
Crude model	1.03 (0.96, 1.10)	1.03 (0.97, 1.09)	Dropped	0.95 (0.88, 1.02)	0.99 (0.93, 1.05)
Multivariable model 1[d]	1.01 (0.95, 1.07)	1.03 (0.97, 1.09)	Dropped	0.95 (0.89, 1.01)	1.00 (0.94, 1.06)
Multivariable model 2[e]	1.01 (0.95, 1.07)	1.03 (0.97, 1.09)	Dropped	0.95 (0.88, 1.02)	1.00 (0.94, 1.06)
**Substitution model D: Moderate-vigorous PA is dropped**					
Crude model	1.08 (1.03, 1.13)**	1.09 (1.04, 1.14)**	1.06 (0.98, 1.13)	Dropped	0.93 (0.89, 0.97)**
Multivariable model 1[d]	1.07 (1.02, 1.12)**	1.07 (1.03, 1.11)**	1.06 (0.99, 1.13)	Dropped	0.94 (0.89, 0.99)*
Multivariable model 2[e]	1.06 (1.01, 1.11)*	1.06 (1.01, 1.11)**	1.05 (0.97, 1.13)	Dropped	0.95 (0.90, 1.00)

Abbreviations: CI, confidence interval; RR, Relative Risk; PA, physical activity. [a]Hypertriglyceridemia is defined as plasma triglycerides concentrations ≥1.7 mmol/L or ≥150 mg/dL, or the use of hypolipidemic agents (fibrates). [b]Light PA (<4.0 METs) includes leisurely stroll or walk. Moderate-vigorous PA (≥4.0 METs) includes faster walking, cross country walking, stair climbing, working in the garden, guided exercises and outdoor sports or at home or at the gym. [c]Total discretionary time= sum of time spent in sleeping, TV-watching, light PA and moderate-vigorous PA. The coefficient for total discretionary time represents the omitted activity component (e.g. sleeping for substitution model A, TV-watching for substitution model B and so on). [d]Multivariable-adjusted model for age (continuous), gender, education level (illiterate/primary education, secondary education and academic/graduate), smoking status (never smoker, past smoker and current smoker), marital status (single/divorced, married and widower), familiar history of coronary heart disease (yes or no) and Mediterranean diet adherence (<8 or ≥9 items). [e]Multivariable-adjusted model for variables in model 1 plus the other four metabolic syndrome individuals components. All models were stratified by recruiting center.*P<0.05, ** P<0.01.

S1 Table 7 Multivariable-adjusted RR (95% CI) for low-HDL-c[a] per 60-min/day substitution in sleeping, watching TV and PA

**Substitution Model** (Substitution of 60 min/day of an activity to replace 60 min/day for one of the remaining activities)	**Sleeping**	**TV-watching**	**Light PA[b]**	**Moderate-vigorous PA[b]**	**Total discretionary time[c]**
**Substitution model A : Sleeping is dropped**					
Crude model	Dropped	0.98 (0.95, 1.01)	0.96 (0.89, 1.02)	0.89 (0.84, 0.94)**	1.03 (1.00, 1.06)
Multivariable model 1[d]	Dropped	0.97 (0.94, 1.00)	0.97 (0.90, 1.04)	0.91 (0.86, 0.96)**	1.02 (0.99, 1.05)
Multivariable model 2[e]	Dropped	0.97 (0.94, 1.00)	0.97 (0.90, 1.04)	0.92 (0.87, 0.97)**	1.02 (0.99, 1.04)
**Substitution model B : TV-Watching is dropped**					
Crude model	1.01 (0.98, 1.04)	Dropped	0.97 (0.91, 1.03)	0.87 (0.84, 0.90)**	1.01 (0.99, 1.03)
Multivariable model 1[d]	1.02 (0.99, 1.05)	Dropped	0.99 (0.93, 1.05)	0.92 (0.88, 0.96)**	1.01 (0.99, 1.03)
Multivariable model 2[e]	1.03 (1.00, 1.06)	Dropped	0.99 (0.93, 1.05)	0.94 (0.90, 0.98)*	1.01 (0.98, 1.03)
**Substitution model C : Light PA is dropped**					
Crude model	1.04 (0.96, 1.12)	1.03 (0.96, 1.10)	Dropped	0.89 (0.83, 0.95)**	0.98 (0.92, 1.04)
Multivariable model 1[d]	1.03 (0.96, 1.10)	1.01 (0.94,1.08)	Dropped	0.90 (0.84, 0.96)**	1.00 (0.94, 1.06)
Multivariable model 2[e]	1.04 (0.97, 1.11)	1.01 (0.95,1.07)	Dropped	0.92 (0.86, 0.98)**	0.99 (0.93, 1.05)
**Substitution model D: Moderate-vigorous PA is dropped**					
Crude model	1.13 (1.07, 1.19)**	1.14(1.09, 1.19)**	1.12 (1.05, 1.19)**	Dropped	0.89 (0.85, 0.94)**
Multivariable model 1[d]	1.10 (1.04, 1.16)**	1.08 (1.03, 1.13)**	1.10 (1.02, 1.18)**	Dropped	0.93 (0.88, 0.98)**
Multivariable model 2[e]	1.08 (1.02, 1.14)**	1.06 (1.01, 1.11)**	1.09 (1.02, 1.16)*	Dropped	0.95 (0.91, 0.99)**

Abbreviations: CI, confidence interval; RR, Relative Risk; PA, physical activity; HDL-c, high density lipoprotein-cholesterol. [a]Low HDL-c is defined as plasma HDL-c concentrations ≤40 mg/dL o ≤1.03 mmol/L in men or ≤50 mg/dL or ≤1.3 mmol/L in women or the use of hypolipidemic agents. [b]Light PA (<4.0 METs) includes leisurely stroll or walk. Moderate-vigorous PA (≥4.0 METs) includes faster walking, cross country walking, stair climbing, working in the garden, guided exercises and outdoor sports or at home or at the gym. [c]Total discretionary time= sum of time spent in sleeping, TV-watching, light PA and moderate-vigorous PA. The coefficient for total discretionary time represents the omitted activity component (e.g. sleeping for substitution model A, TV-watching for substitution model B and so on. [d]Multivariable-adjusted model for age (continuous), gender, education level (illiterate/primary education, secondary education and academic/graduate), smoking status (never smoker, past smoker and current smoker), marital status (single/divorced, married and widower), familiar history of coronary heart disease (yes or no) and Mediterranean diet adherence (<8 or ≥9 items). [e]Multivariable-adjusted model for variables in model 1 plus the other four metabolic syndrome individuals components. All models were stratified by recruiting center.
* P<0.05, ** P<0.01.
